# Cannabinoids in the landscape of cancer

**DOI:** 10.1007/s00432-021-03710-7

**Published:** 2021-07-14

**Authors:** Nagina Mangal, Simon Erridge, Nagy Habib, Anguraj Sadanandam, Vikash Reebye, Mikael Hans Sodergren

**Affiliations:** 1 Medical Cannabis Research Group, Department of Surgery and Cancer, Imperial College London, Hammersmith Campus, London, W12 0HS UK; 2 Systems and Precision Cancer Medicine Team, Division of Molecular Pathology, Institute of Cancer Research, London, SM2 5NG UK

**Keywords:** Cannabinoids, Cancer, Cannabidiol, Tetrahydrocannabinol, Cannabinoid receptors, Endocannabinoid system

## Abstract

**Introduction:**

Cannabinoids are a group of terpenophenolic compounds derived from the *Cannabis sativa* L. plant. There is a growing body of evidence from cell culture and animal studies in support of cannabinoids possessing anticancer properties.

**Method:**

A database search of peer reviewed articles published in English as full texts between January 1970 and April 2021 in Google Scholar, MEDLINE, PubMed and Web of Science was undertaken. References of relevant literature were searched to identify additional studies to construct a narrative literature review of oncological effects of cannabinoids in pre-clinical and clinical studies in various cancer types.

**Results:**

Phyto-, endogenous and synthetic cannabinoids demonstrated antitumour effects both in vitro and in vivo. However, these effects are dependent on cancer type, the concentration and preparation of the cannabinoid and the abundance of receptor targets. The mechanism of action of synthetic cannabinoids, (−)-trans-Δ^9^-tetrahydrocannabinol (Δ^9^-THC) and cannabidiol (CBD) has mainly been described via the traditional cannabinoid receptors; CB_1_ and CB_2_, but reports have also indicated evidence of activity through GPR55, TRPM8 and other ion channels including TRPA1, TRPV1 and TRPV2.

**Conclusion:**

Cannabinoids have shown to be efficacious both as a single agent and in combination with antineoplastic drugs. These effects have occurred through various receptors and ligands and modulation of signalling pathways involved in hallmarks of cancer pathology. There is a need for further studies to characterise its mode of action at the molecular level and to delineate efficacious dosage and route of administration in addition to synergistic regimes.

## Introduction

Since time immemorial, the Cannabis plant has been used as a source of fibre, herbal remedy, medicinal and religious purposes (Kalant 2001; Goncalves et al. 2020). In the mid-nineteenth century, O’Shaughnessy and Moreau reported positive effects of cannabis on muscle spasms, vomiting, convulsions, rheumatism, tetanus, and rabies (O’Shaughnessy 1843; Zuardi 2006). However, during the twentieth century, its utilisation in Western medicine started to decline as a result of political prejudices and economic interests rather than scientific or medical reasons (Zuardi 2006). Over recent years, cannabis and its derivatives have been used for treating chemotherapy induced nausea and vomiting, epilepsy and multiple sclerosis amongst other indications (Parker et al. 2011; Kleckner 2019). Increasing data from and in vivo studies have started to show evidence of cannabis in modulating signalling pathways involved in cancer cell proliferation, autophagy, apoptosis and inhibition of angiogenesis and metastasis (Velasco et al. 2016). Emerging reports have also indicated synergistic effects of cannabinoids in combination with antineoplastic drugs (Moreno et al. 2019; Dariš et al. 2019; Fogli et al. 2006; Velasco et al. 2012).

**Fig. 1:**
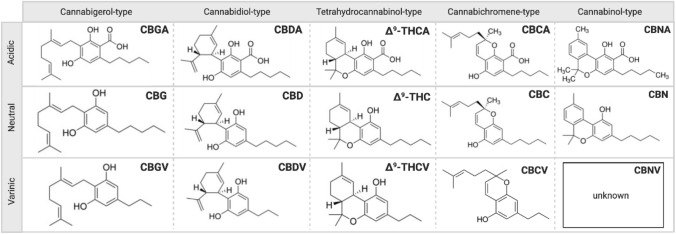
The chemical structures of Cannabigerol (CBG), Cannabidiol (CBD), Tetrahydrocannabinol (Δ^9^-THC), Cannabichromene (CBC) and Cannabinol (CBN)-type neutral, varinic and acidic phytocannabinoids. More than 120 phytocannabinoids have been isolated from *Cannabis sativa* L*.* which can be distinguished into eleven chemical subtypes (Gonçalves et al. 2020; ElSohly 2017). Their common chemical features include a dibenzopyran ring and a hydrophobic alkyl chain (Morales et al. 2017). Aside from Δ^9^-THC and CBD, there has been a current focus on the therapeutic properties of some minor, varinic and acidic cannabinoids (Andre et al. 2016; Franco et al. 2020). Created with BioRender.com

**Fig. 2:**
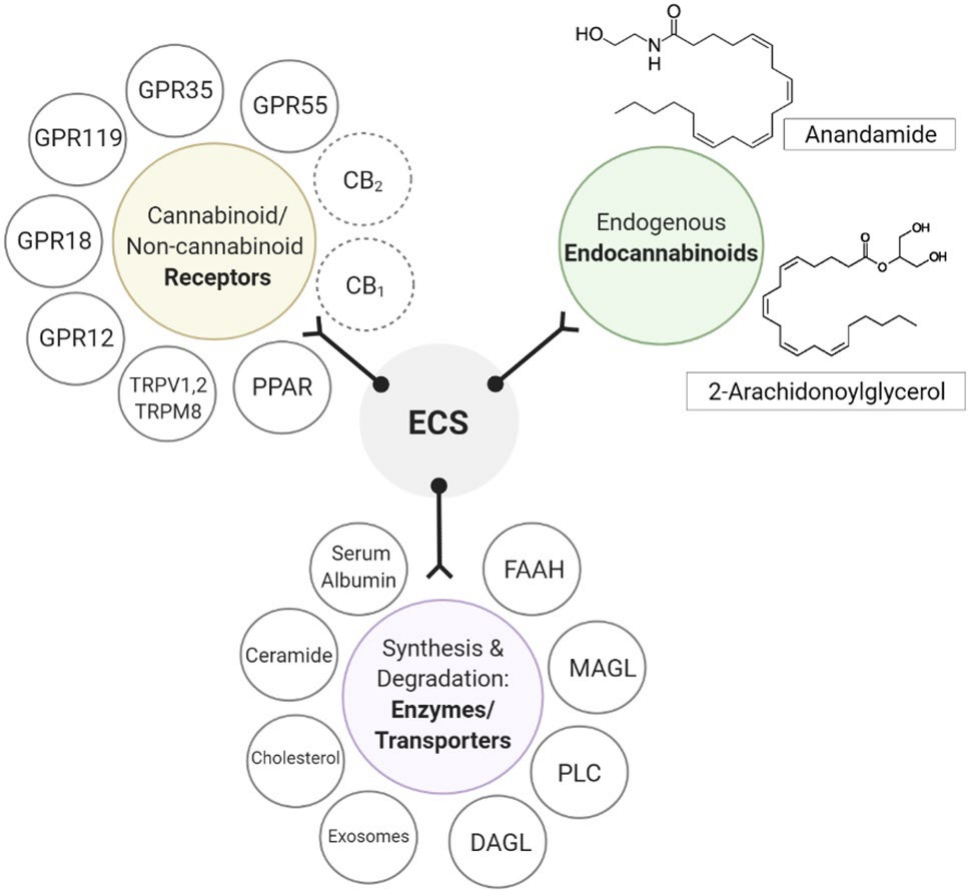
Overview of the components of the endocannabinoid system (ECS) which include endogenous endocannabinoids; Anandamide (AEA) and 2-Arachidonoylglycerol (2-AG), its major receptors classified into cannabinoid receptors 1 and 2, and non-cannabinoid receptors; GPR55, GPR35, GPR119, GPR18, GPR12, ion channels including transient receptor potential cation channel subfamily members; TRPM8, TRPV1, TRPV2, peroxisome-proliferator-activated receptors (PPAR). A third component of the system are its enzymes/transporters responsible for the synthesis and degradation of endocannabinoids including serum albumin, ceramide, cholesterol, diacylglycerol lipase (DAGL), phospholipase C (PLC), monoacylglycerol lipase (MAGL), fatty acid amide hydrolase (FAAH). Created with BioRender.com

The cannabis plant has been termed as a “storehouse” of several pharmacologically relevant compounds (Andre et al. 2016). The unique qualities of each cannabis variety or chemovar are the result of varying concentrations of numerous classes of bioactive molecules, most notably, cannabinoids as shown in Fig. 1, terpenoids and flavonoids (Chakravarti et al. 2014). Cannabinoids interact directly with cannabinoid receptors, which include G-protein coupled receptors (cannabinoid receptor 1, CB_1_ and cannabinoid receptor 2, CB_2_), ligand-gated ion channels (i.e. vanilloid cell surface channels) and nuclear receptors (i.e. peroxisome proliferator-activated receptor gamma, PPARγ) (Moreno et al. 2019; Śledziński et al. 2018) comprising the endogenous endocannabinoid system (ECS) (Zou and Kumar 2018). Three major classifications of cannabinoids include phytocannabinoids (plant-based), such as Δ^9^-tetrahydrocannabinol (Δ^9^-THC) and cannabidiol (CBD), endocannabinoids (or endogenous cannabinoids) which include anandamide (AEA) and 2-arachidonolyglycerol (2-AG) and synthetic cannabinoids that mimic the cannabinoid groups (1) and (2) (Pertwee 2006; Lu and Mackie 2016). Endocannabinoids play a crucial role in mediating physiological functions including metabolic, cardiovascular regulation, reproduction, inflammatory response, immune system and analgesia (Guindon and Hohmann 2012; Kaur et al. 2016). AEA and 2-AG are degraded by fatty acid amide hydrolase (FAAH) and monoacylglycerol lipase (MAGL) enzymes (Pisanti et al. 2013). Modulation of their activity may have potential therapeutic implications and inhibitors are under active investigation as pharmaceuticals. Synthetic cannabinoids have been studied extensively and some have been shown to be highly bioactive than their natural counterparts, some common ones include WIN55, 212–2 (potent CB_1_ receptor agonist), JWH-018, JWH-073, JWH-133 (CB receptor agonists) and SR141716 or Rimonabant (CB_1_ receptor antagonist) (Morales et al. 2017), overview shown in Fig. 2.

Several studies have reported the varying affinities of phytocannabinoids for the classical CB_1_ and CB_2_ receptors with agonistic and antagonistic behaviours (Morales et al. 2017; Zhao and Abood 2013). However, it is now emerging that cannabinoids can interact with multiple orphan G-protein coupled receptors (GPCRs) including GPR12, GPR18, GPR35, GPR55, GPR119, opioid and serotonin receptors (Morales et al. 2017; Zhao and Abood 2013; Console-Bram et al. 2014; Brown et al. 2017; Soderstorm et al. 2017; Ferro et al. 2018; Guerrero-Alba 2019). The interaction of GPCRs is crucial for maintaining the ECS as it allows the production of endocannabinoids from cells through activation of *G*_q/11_ or *G*_s_ proteins causing the activation of the cannabinoid receptor (Gyombolai et al. 2012). Furthermore, the downstream receptor-mediated effects of endocannabinoids also contribute to the plasticity of the ECS (Lu and Mackie 2016).

Since the first report of cannabinoids anticancer effects (Munson et al. 1975), there have been many studies investigating phytocannabinoids, endogenous and synthetic ones in multiple cancer models. Various signalling pathways and changes to internal conditions which favour antitumour activity by cannabinoids have been observed. CBD amongst other cannabinoids has shown to increase the de novo synthesis of ceramide through upregulation of a plethora of enzymes each catalysing specific biochemical steps. Ceramide synthases are one of the major group of enzymes involved and reports have revealed an upregulation of its six isoforms; CerS 1–6 (Ceramide Synthases 1–6) in cancer via cannabinoids (Gomez et al. 2002; Gustafsson et al. 2009; Schiffman et al. 2009). However, it is not clear whether specific isoform(s) upregulation correlates to the cancer type and whether this is also specific to the type of cannabinoid. An interesting finding from a report has shown siRNA-induced knockdown of ceramide synthase 1 (CerS1 isoform) prevented gemcitabine-induced caspase 9 activation (Senkal et al. 2007; Levy and Futerman 2010). This could be explored further when considering cannabinoids action synergistically with chemotherapy drugs as ceramide may have the ability to sensitize the cancer cells to chemotherapy agents. Another major area of cannabinoids action has been through modulating the cell cycle. In a recent report in gastric cancer cells, CBD-induced cell cycle arrest at the *G*_0_–*G*_1_ phase and retardation in this phase corresponded to a reduction in CDK2/cyclin E protein levels (Zhang et al. 2019). Apoptotic changes are prevalent in cannabinoids mechanism of action which include morphological changes to the cells and cytoplasmic vacuolization, an increase in cleaved caspase-3 and -9 levels and activation of the mitochondrial apoptotic pathway (Zhang et al. 2019; Schoeman et al. 2020). Endoplasmic reticulum (ER) stress which occurs following ceramide synthesis causes downstream apoptotic changes and increases in proapoptotic proteins, such as BAD and Bax, also resulting in an increase in reactive oxygen species (ROS) signalling (Zhang et al. 2019). Δ^9^-THC in glioma cells has shown to induce upregulation of the p8 protein (involved in ER stress and metastasis) via de novo synthesis of ceramide (Carracedo et al. 2006). From the literature available, it is evident that there is an interplay between cannabinoids downstream effects.

Overall cannabinoids induce apoptosis to inhibit proliferation, downregulate the vascular endothelial growth factor (VEGF) pathway affecting angiogenesis and dampen metastasis by inhibiting cell adhesion and migration through modifying matrix metalloproteinase 2, 9 (MMP2, 9), tissue inhibitor of matrix metalloproteinases 1 (TIMP1), inhibitor of DNA binding 1 (ID1) and inducing ER stress (Velasco et al. 2016). Cancer cells do not exist in isolation and the tumour microenvironment (TME) has also been an imperative target for cancer therapy as it can influence the propensity for tumour growth, metastasis and resistance to therapy. The TME is composed of a host of factors including cancer-associated fibroblasts (CAFs), immune and inflammatory cells, lymph and blood vasculature, neuroendocrine cells, and extracellular matrix (ECM) (Wang et al. 2017). Cancer stem cells (CSCs), a subpopulation of stem cells expressing CD44, CD24 and CD133, are tumorigenic with demonstrated resistance to certain chemotherapeutics and also play a role in metastasis (Yu et al. 2012). Reports have shown the involvement of cannabinoids in inhibiting CAFs and CSCs in prostate and breast cancer models (Sharma et al. 2014; Mohammadpour et al. 2017; Pietrovito et al. 2020). The aforementioned effects, however, occur at varying degrees which depend on the cancer cell line, the expression levels of cannabinoid receptors, the type of cannabinoid compound and dosage.

The aim of this review is to analyse pre-clinical work and outline previous and forthcoming clinical research studies exploring cannabinoids in cancer treatment. Below, we outline the research encompassing endogenous and non-endogenous cannabinoids in which we review the proposed mechanisms of action culminated from studies into various cancers and discuss the need for more clinical studies to explore the possible therapeutic efficacy of cannabinoids as a possible treatment for cancer.

## Method

### Research question

This narrative review was conducted of available literature reporting the treatment effects of all cannabinoids as either a single agent or co-administered with other antitumour therapies in all cancer types. The aim of this review is to analyse and evaluate pre-clinical and clinical research determining the use of cannabinoids as a potential anti-cancer therapy.

### Search strategy and inclusion criteria

A broad electronic search was conducted on Google Scholar, MEDLINE, PubMed and Web of Science articles published in English between 1st January 1970 and 30th April 2021. Investigations of cannabinoids use in oncology clinical trials were searched using the database, clinicaltrials.gov.uk with the key words; “Cannabinoids and Cancer”, “Cannabis and Cancer”, “Tetrahydrocannabinol and Cancer”, “CBD and Cancer” and “THC and Cancer”. The literature search was performed by two independent researchers (N.M. and S.E.) and if any discrepancies were identified then these were resolved by a senior author (M.S.). The reference lists of all publications were screened for further relevant references. The free text search included articles citing both original research and literature reviews. Inclusion criteria encompassed all reports identifying cannabinoids use in pre-clinical cancer models which includes in vitro, in vivo and in ovo experimental models, as well as clinical research. In addition, reports of potential mechanisms of action and signalling pathways involved were also included. Where literature reviews were identified, the relevant cited studies were also identified and included for de novo analysis.

### Data extraction and presentation

Two independent researchers (N.M. and S.E.) performed the data extraction. Primary research papers reporting half maximal inhibitory concentration (IC_50_) and concentrations where the described effects were observed in pre-clinical cancer models were included in separate tables for in vitro and in vivo investigations. Concentration values are presented as micro-molar concentrations (μM) with their standard deviation (S.D.), standard error (S.E), or range except when unreported in the original study.

## Results

### Mechanism of action and signalling pathways

**Fig. 3:**
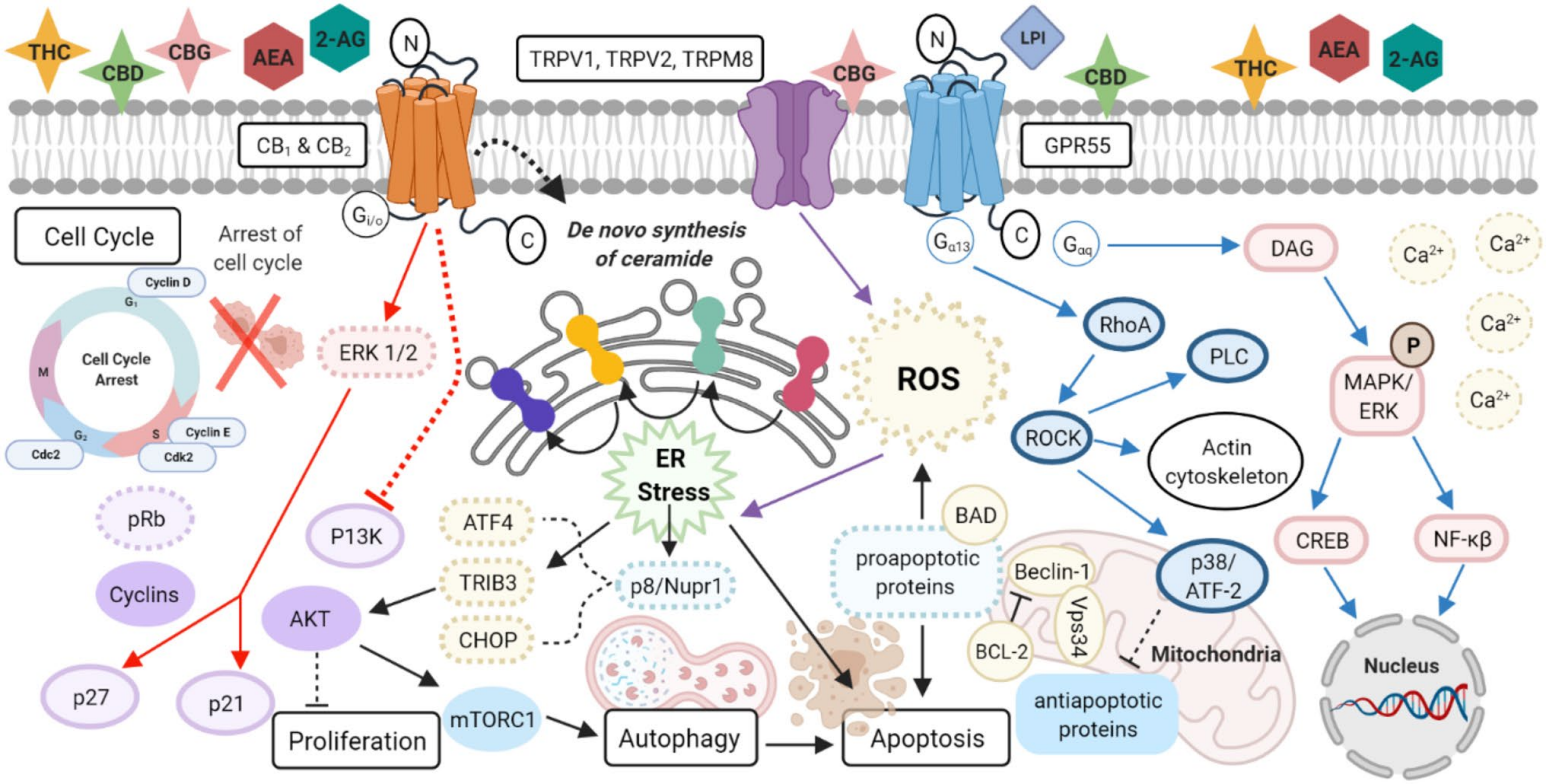
Overview of the downstream activation and crosstalk of signalling pathways of cannabinoid and non-cannabinoid receptors. Activation of the cannabinoid receptors CB_1_ and CB_2_ (red arrows) via cannabinoids stimulate ERK1/2 signalling which activates p27 and p21 causing a decrease in cyclins D and E, cdc2 and cdk2 through an increase in pRb, leading to cell cycle arrest. Inhibition of the P13K pathway leads to a decrease in Akt which inhibits cell proliferation. Biosynthesis of ceramide takes place at the endoplasmic reticulum through a series of biochemical steps involving many enzymes which help to convert dihydroceramides (DhCers) into ceramide. An increase in ceramide level in turn increases the stress protein p8/Nupr1 and TRIB3 which activates upregulation of ATF4 and CHOP proteins. A decrease in Akt leads to a downregulation in mTORC1 signalling causing autophagy. Activation of TRPM8 (purple arrows) leads to an increase in ROS production which also induces ER stress. Stimulation of non-cannabinoid receptor GPR55 (blue arrows) through LPI via the subunit Gα_q_ subunit stimulates the production of PLC to release Ca^2+^ and DAG which leads to the activation of MAPK/ERK signalling. This causes gene transcription by activation of transcription factors CREB and NF-κß. Gα_12/13_ subunit activates the RhoA/ROCK pathway which regulates PLC, actin cytoskeleton and p38/ATF2 activity. ATF2/p38 inhibits antiapoptotic proteins and enhances the interaction between Beclin-1 and Vps34 which is also inhibited by BCL-2 further enhancing ROS production by activation of the intrinsic apoptotic pathway (Velasco et al. 2012, 2016).Created with BioRender.com. *TRPV1,2* transient receptor potential cation channel subfamily V member 1,2, *TRPM8* transient receptor potential cation channel subfamily members (melastatin) 8, *GPR55* orphan G-protein coupled receptor 55, *ROS* reactive oxygen species, *ER* endoplasmic reticulum, *p8* protein p8 (Nuclear Protein 1, NUPR1), *CHOP* CCAAT/-enhancer-binding protein homologous protein, *ATF4* activating transcription factor 4, *TRIB3* tribbles pseudokinase 3, *Akt* protein kinase B, *mTORC1* mammalian target of rapamycin C1, *p21* cyclin-dependent kinase inhibitor 1, *p27* cyclin-dependent kinase inhibitor 1B, *CDK* cyclin-dependent kinase, *pRb* retinoblastoma protein; Nuclear factor-kappaß (NF-κß), *LPI* Lysophosphatidylinositol, *DAG* diacylglycerol, *BAD* BCL2-associated agonist of cell death, *ROCK* rho-associated protein kinase, *PLC* phospholipase C

The ECS is a complex system composed of different ligands, receptors and ion channels resulting in many signalling pathways subject to modulation from external cannabinoids as shown in Fig. 3. It is therefore no surprise that there remains ambiguity in its precise role within cancer pathophysiology (Wu 2019). Many pre-clinical studies and histological analysis of patient tumours, suggest that an upregulation in the CB_1_ and CB_2_ receptors, endogenous ligands and over-activation of the ECS correlates with more aggressive tumours (Dariš et al. 2019) although other reports have concluded the contrary (Jung et al. 2013; Tutino et al. 2019). Cancer is a heterogenous disease and current evidence should be interpreted on the basis that different tumour types have been shown to exhibit various levels of CB receptors as well as ECS components. The role of the endogenous endocannabinoids and CB receptors within each cancer system is specific to the underlying cancer, therefore conflicting data can be presented across different cancers. It has also been reported that some cannabinoids have shown oncological effects independent of known CB receptors (Moreno et al. 2019; Fogli et al. 2006) implying that there may be undiscovered cannabinoid receptors implicated in cancer pathophysiology.

The characterisation of cannabinoids mechanism of action has been discerned from in vitro and in vivo studies. Reports of their oncological effects have been observed through modulating the hallmarks of cancer (Hanahan and Weinberg 2000, 2011) whilst ∆^9^-THC trends in inducing apoptosis and cytotoxicity through CB receptor-dependent pathways; CBD exhibits its activity via orphan GPCRs and non-GPRCs-mediated signalling (Velasco et al. 2012, 2016; Afrin et al. 2020).

Studies have reported positive upregulation of ceramide sphingolipid metabolism, leading to the subsequent arrest of the cell cycle and apoptosis via downstream activation of signals through extracellular regulated kinase (ERK) upon cannabinoid action (Calvaruso et al. 2012). Additional studies have also concluded ∆^9^-THC’s role in regulating sphingolipid metabolism via serine palmitoyl transferase (SPT) (Śledziński et al. 2018) and recent reports have concluded other enzymes of the metabolism of sphingolipids to be regulated by cannabinoids (Shaw et al. 2018). Dihydroceramides which are metabolic intermediates of the de novo synthesis pathway have been involved in the mechanisms of promoting autophagy-mediated cancer cell death (Hernández-Tiedra et al. 2016). ∆^9^-THC increases the dihydroceramide:ceramide ratio in the endoplasmic reticulum of glioma cells causing pre-apoptotic changes (Hernández-Tiedra et al. 2016).

Activation of the CB receptors causes the induction of the ER stress-related response and promotes the upregulation of the transcription factor p8 (Nupr1), this further simulates the following transcription factors, activating transcription factor 4 (ATF-4), C/EBP-homologous protein (CHOP) and pseudokinase tribbles-homologue 3 (TRIB3) (Velasco et al. 2016). The inhibitory interaction of TRIB3 and a pro-survival kinase Akt is favoured which leads to the inhibition of the mammalian target of rapamycin target 1 (mTORC1) favouring cell autophagy. Autophagy is upstream of apoptosis in cannabinoid-induced cell death as shown in studies where blocking autophagy prevented cannabinoid-induced apoptosis (Salazar et al. 2009; Vara et al. 2011). An increase in ceramide level has also been associated with ER stress in cannabinoid-induced apoptosis in tumour cells (Salazar et al. 2009). In addition, other environmental stimuli may also promote ER stress which can lead to the activation of the apoptotic pathway. These include a decrease in intracellular Ca^2+^, viral infections, chemotherapy agents and oxidative stress (Schröder and Kaufman 2005; Śledziński et al. 2018).

The mitogen-activated protein kinase (MAPK) pathway has also been reported in numerous studies to be involved in cannabinoid response. Serine/threonine protein kinases are mainly involved in this pathway and act to convert extracellular stress into different cellular responses including, cell cycle arrest, apoptotic cell death and cytokine production via phosphorylation. The involvement of the MAPK pathway in cancer is complex as its response to different stimuli can produce conflicting outcomes. Brief activation of the ERK cascade leads to cell survival and proliferation, whilst chronic activation is pro-apoptotic (Howlett 2005; Javid et al. 2016).

CBD has been demonstrated to affect a diverse set of cellular targets. First, it inhibits FAAH and FABP (Fatty Acid-Binding Protein). FAAH is responsible for the breakdown of anandamide, whilst FABP aids the transport of anandamide to from extracellular spaces to intracellular targets, such FAAH or nuclear PPAR. Both effects result in indirect activation of CB_1_ and CB_2_ receptors through increased extracellular concentration of anandamide (Lee et al. 2007; Pistis and O’Sullivan 2017). Second, CBD activates the 5-HT_1A_ serotonin receptor, PPARγ and the transient receptor potential cation subfamily channels; TRPV1, TRPV2 and TRPA1. CBD is also an antagonist of GPR55, transient receptor potential cation channel subfamily M member 8 (TRPM8) and T-type Ca^2+^ channels. Finally, CBD has also been reported to inhibit adenosine reuptake via multiple proposed mechanisms (Lee et al. 2007; Ibeas Bih et al. 2015; McPartland 2018). Antagonization of GPR55 via CBD has been reported to reduce proliferation of pancreatic tumour cells and its activation has been reported to lead to metastasis in triple-negative breast cancer when stimulated by LPI (Zhao and Abood 2013; Ferro et al. 2018; Andradas et al. 2016; Falasca and Ferro et al. 2018; Pellati et al. 2018). Below we summarise pre-clinical studies which include both in vitro and in vivo experimental results in various cancer models with summaries included in Tables 1 and 2.

**Table 1:** Pre-clinical in vitro studies encompassing various cannabinoids in cancer models

	Cancer Cell line	Cannabinoid (s)	Inhibitory concentrations	In vitro actions	References
PDAC	MIA PaCa2, PANC-1, Capan-2, BxPC-3	Δ^9^-THC, SR141716, SR144528	0–5 µM	Apoptosis via CB_2_ and p8, ATF4 and TRIB3 and caspase-3 activation	Carracedo et al. (2006)
	AsPC-I, HPFA-II, PANC-I, BxPC-3	CBD	0–10 µM	Antiproliferative effects via GPR55	Ferro et al. (2018)
BRAIN	Human Glioblastoma, U373-MG	Δ^9^-THC, AEA, HU-210, WIN 55,212–2	100 nM–10 µM	Accelerated cell proliferation via EGFR and MMP	McAllister et al. (2011)
	Human Glioblastoma, U878MG, U373MG	CBD, SR141716, SR144528	5–40 µM	Antiproliferative effects correlated to induction of apoptosis	Singer et al. (2015)
	Human Glioblastoma multiforme, SF126, U87-MG, U251, SF188, U373-MG, Human GBM cultures	Δ^9^-THC, WIN 55,212–2	0.1 nM-2 µM	Antiproliferative effects and increase of apoptosis	Ellert-Miklaszewska et al. (2021)
	Rat C6 glioma cells	WIN 55,212–2, WIN 55,212–3	1–30 µM	Cell viability reduction, morphological changes to cells	Matas et al. (2007)
	Rat C6 glioma cells	Δ^9^-THC, CBD, CBD-A, CBG, CBC, AM251, JWH-133, AM630, SR141716A, SR144528	0–50 µM	CBD most potent. CBD, CBG and CBD-A activated TRPV1	Ligresti et al. (2006)
	Murine Neuroblastoma, N18TG2	AEA	1–5 µM	Apoptosis and decrease in cleavage of PARP-1	Marcu et al. (2010)
	Human Astrocytoma, U87MG	Δ^9^-THC, SR141716	1–10 µM	Apoptosis and autophagy via ER stress	Salazar et al. (2009)
	Human Glioma cancer, U251, SF126, U87	Δ^9^-THC, CBD	0.1–10 µM	Inhibition of cell proliferation, apoptosis	Qamri et al. (2009)
	Human Glioblastoma, U87-MG, T98G	CBD	0–20 µM	Decrease in cell invasion via MMP-9, TIMP-1, TIMP-4, u-PA, PAI-1, VEGF	Solinas et al. (2013)
	Human Glioma, T98G, U87MG, Murine Glioma, GL261	CBD, Δ^9^-THC (Pure and BDS)	0–20 µM	Increase in radiosensitivity associated with increase in apoptosis and autophagy	Scott et al. (2014)
	Human Glioblastoma, U251, 3832, 387 Primary glioma stem cells (GSC) lines	CBD	0–5 µM	Activation of p-p38 pathway, downregulation of key stem cell regulators; Sox2, Id1 and p-STAT3	Singer et al. (2015)
	Human Neuroblastoma, SK-N-SH, IMR-32, NUB-6 and LAN-1	Δ^9^-THC, CBD	0–50 µg/mL	Cell viability reduction and apoptosis	Fisher et al. (2016)
	Human Glioblastoma, U87MG, Glioblastoma patient derived stem cell like cells (GIC)	Δ^9^-THC, CBD	0–5 µM	Very significant reduction of the GIC population, induction of apoptosis	López-Valero et al. (2018)
	Human Glioma cells, U87MG (U87), A172, SW1783, U373MG (U373), T98G (T98), SW1088, and LN405	Δ^9^-THC, CBD, SR141716, SR144528	0.9–3 µmol/L	Reduction in cell viability and induction of apoptosis and autophagy	Torres et al. (2011)
	Human Glioma cells, GOS3, U87 MG (U87), A172, SW1783, U118 MG (U118), U373 MG (U373), T98G (T98), SW1088, CCF-STTG1 (CCF) and LN405	Δ^9^-THC, SR141716, SR144528	0–2.5 µM	Sensitive and resistance cell line determined via reduction in cell viability Increased Mdk expression confers resistance of glioma cells to Δ^9^-THC pro-autophagic and antitumoural action	Lorente et al. (2011)
BREAST	Human Breast adenocarcinoma, MDA-MB-231, MCF-7, murine mammary carcinoma, 4T1	Δ^9^-THC	0- 20 µM	No decrease observed in cell viability for all cell lines and low level of cannabinoid receptors	McKallip et al. (2005)
	Human Breast adenocarcinoma, EVSA-T	Δ^9^-THC	3 and 5 µM	Antiproliferative effects rely on JunD activity and participation of p8	Caffarel et al. (2008)
	Human Breast adenocarcinoma, MDA-MB-231, T47D, murine breast cancer, TSAE-1	Met-F-AEA, SR141716A	2.5–20 µM	Reduction in cell viability in dose-dependent manner and decrease of tyrosine phosphorylation of FAK and Src	Santoro et al. (2009)
	Human Breast adenocarcinoma, MDA-MB-231, T47D, MCF-7	SR141716	0.1–1 µM	Cell cycle arrest, decreased expression of cyclins D and E Antiproliferative effect requires lipid raft/caveolae integrity to occur	Sarnataro et al. (2005)
	Human Breast adenocarcinoma, EVSA-T, MDA-MB-231, MDA-MB-468, SKBR3, MCF-7, T-47D	Δ^9^-THC, SR141716, SR144528	1–12 µmol/L	Reduction in cell proliferation via the CB_2_ receptor, cell cycle arrest, induction of apoptosis	Caffarel et al. (2006)
	Human Breast adenocarcinoma, MDA-MB-231, MCF-7	Δ^9^-THC, CBD, CBG, CBC, AM251, JWH-133, AM630, SR141716A, SR144528	0–50 µM	CBD apoptotic effect via activation of the CB_2_ receptor and TRPV1	Ligresti et al. (2006)
	Human Breast adenocarcinoma, MDA-MB-231, MDA-MB-231-Luc, MDA-MB-468	WIN 55,212–2, JWH-133, AM251, SR144528	0–10 µM	All cell lines express both CB_1_ and CB_2_ receptors Inhibition of cell proliferation and migration via COX-2 signalling and apoptosis	Hirao-Suzuki et al. (2020)
	Human Breast adenocarcinoma, MDA-MB231 Murine mammary carcinoma, 4T1.2	CBD	1.5 µM	Inhibition of cell proliferation and invasion through modulation of ERK and ROS, downregulation of Id-1 expression and upregulation of Id-2	Nallathambi et al. (2018)
	Human Breast adenocarcinoma, MDA-MB-231, SKBR3, MCF-7, ZR-75–1	CBD, AM251, AM630, Capazepine	0–10 µM	Decrease in cell viability, autophagy and apoptosis via ER stress, inhibition of Akt, mTOR signalling	Lin et al. (2019)
	Human Breast adenocarcinoma, SUM159, MDA-MB-231-SCP2, MVT-1, murine mammary carcinoma, 4T1.2	CBD	3–15 µM	Cell proliferation decreased, inhibition of the epidermal growth factor (EGF)-induced cell proliferation, migration, and invasion	Grimaldi et al. (2006)
	Human Breast adenocarcinoma, MCF-7, Murine mammary carcinoma, 4T1	JWH-015, SR141716, SR144528	0–10 µM	Decrease in cell viability, apoptosis and reduced ERK1/2 levels, effects were dependent in a non-Gαi -mediated, calcium-dependency	McAllister et al. (2011)
	Human Breast adenocarcinoma, MDA-MB-231	AEA, AM251	0–0.5 µM	Reduction in CD44^+^/CD24^−/low^/ESA^+^ cancer stem cell (CSC) invasiveness	Mohammadpour et al. (2017)
	Human Breast adenocarcinoma, MDA-MB-231	CBDA, GSK0660, GW501516, ST-247	1–50 µM	CBDA inhibits PPARβ/δ mediated transcriptional activation and AP-1	Gazzerro et al. (2010)
	Human Breast Cancer, MDA-MB-231, MCF-7	CBD	1–50 µM	Co-administration of CBD_sol_ and paclitaxel or docetaxel showed a synergistic effect	Fraguas-Sánchez et al. (2020)
GASTROINTESTINAL	Human Colon cancer, DLD-1, CaCo-2, SW620	SR141716	0.1–20 µM	Reduction in cell proliferation and cell cycle arrest	Aviello et al. (2012)
	Human Colon adenocarcinoma, Caco-2, HCT 116	CBG, AM251, AM630, AMTB (TRPM8 antagonist), CBD, CBDV, CBC	1–50 µM	Apoptosis, increase in ROS production and upregulation of CHOP expression	Borelli et al. (2014)
	Human Colorectal carcinoma, DLD-1, HCT116	CBD BS (botanical substance), CBD, AM630, SR141716, SR144528	0.3–5 µM	Antiproliferative effects, no effect on cell viability	Romano et al. (2014)
	Human Colorectal cancer, Caco-2	CBD	0.1–10 µM	PhysO_2_ cells significantly more sensitive to antiproliferative effects of CBD than AtmosO_2_	Macpherson et al. 2014
	Human Colon cancer, DLD-1	SR141716	0.1–10 µM	Inhibition of cell proliferation at higher concentrations	Gazzerro et al. (2010)
	Human Colon cancer, SW480	CBD, WIN 55,212–2	0–15 µM	Induction of cellular ACPP, DUSP1, DUSP10, cleavage of PARP, Apoptosis	De Petrocellis et al. (2013)
	Human colorectal carcinoma, Caco-2, HCT116	CBD, SR141716, AM251, SR144528, AM630, GW9662, Capsazepine	0.01–10 µM	Reduction in cell viability and expression of phospho-Akt	Aviello et al. (2012)
	Human Colon cancer, HCT116, SW48	SR141716	0–20 µM	Inhibition of cell growth, increase of caspase-3 and cleavage of PARP	Proto et al. (2017)
	Human Colon cancer, HCT116 and DLD-1 Organoids	SR141716	0.1–20 µM	Reduction in colon CSCs proliferation and tumour differentiated cells	Fiore et al. (2018)
	Human Hepatocellular carcinoma, HepG2, HuH-7	Δ^9^-THC, JWH-015, SR141716, SR144528	1–8 µM	Reduction in cell viability occurred via CB_2_ receptor and autophagy	Vara et al. (2011)
	Human Hepatocellular carcinoma, BEL7402	WIN 55, 212–2, AM630, JWH-015	0, 5 or 10 µM	CB_2_ mediated downregulation of phosphorylated ERK1/2	Xu et al. (2016)
	Human Gastric adenocarcinoma, AGS	AEA, Meth-AEA (R- ( +)), CP 55,940	0.5–5 µM	Concentration-dependent effects in cell morphology and loss changes	Ortega et al. (2016)
	Gastric cancer, SGC7901, AGS cells	WIN 55,212–2	5 µM	Inhibition of cell migration, invasion and EMT	Xian et al. (2016)
PROSTATE	Human Prostate Cancer, PC-3	Δ^9^-THC, AM251, WIN55,212–2	0.5–10 µM	Reduction in cell viability and apoptosis	Ruiz et al. (1999)
	Human Prostate Cancer, LNCaP, DU145, PC-3	AEA	1–10 µM	Decrease of EGFR levels in all cell lines via CB_1_ leading to an inhibition of EGF-stimulated growth	Mimeault et al. (2003)
	Human Prostate Cancer, LNCaP	MET-AEA, HU-210, JWH-015, SR141716, SR144528	0.05–5 µM	Involvement of PI3K pathway and modification of androgen receptor expression	Sanchez et al. (2003)
	Human Prostate carcinoma, LNCaP, PC3	WIN-55,212–2, SR141716, SR144528	1–30 µM	Induction in p27/KIP1 and downregulation in cyclin and CDK levels. Upregulation of ERK1/2 and inhibition of PI3k/Akt pathways	Sarfaraz et al. (2006)
	Human Prostate cancer, LNCaP, 22RV1, DU-145, PC-3	CBC, CBD, CBG, CBN, CBDA, CBGA, CBDV, CBGV, THC, THCA, THCV, THCVA	1–10 µM	Decrease in cell viability and activation of the intrinsic apoptotic pathway	De Petrocellis et al. (2013)
	Human Prostate adenocarcinoma, PC-3, Primary cultures; BPH, LGG, HGG, PrC	AEA, 2-AG, Methanandamide (AM-356), SR141716	2.5, 5 and 10 µM	Cell cycle arrest and induction of apoptosis	Orellana-Serradell et al. (2015)
	Human Prostate cancer, LNCaP, PC-3	WIN 55,212–2, SR141716, SR144528	0- 10 µM	WIN prevents neuroendocrine differentiation by downregulation of PI3K/Akt/mTOR signalling	Morell et al. (2016)
LUNG	Human Lung carcinoma, NCI-H292	Δ^9^-THC, AEA, HU-210, WIN 55,212–2	0.1–10 µM	Increase in cell proliferation dependent on EGFR and MMP	Hart et al. (2004)
	Human NSCLC, EGF-induced, A549, SW-1573	Δ^9^-THC	1–20 µM	Apoptosis and inhibition of proliferation via EGF-induced phosphorylation of ERK1/2, JNK1/1 and Akt	Preet et al. (2008)
	Human Lung adenocarcinoma, A549, H460 Primary non-small-cell lung carcinoma cells	CBD, AM251, AM630, Capsazepine, NS-398	0–10 µM	Decrease in the viability of the cells and upregulation of COX-2 and PPAR-γ expression, PGE_2_, PGD_2_, and 15d-PGJ_2_	Ramer et al. (2013)
	Human NSCLC; A549 (epithelial), CALU1 (mesenchymal)	JWH-015, SR144528	0–5 µM	Decreased migratory and invasive abilities via reduction in FAK, VCAM1, MMP2	Ravi et al. (2016)
	Human Lung cancer; A549	WIN 55,212–2	5–20 µM	Decline in cell viability due to apoptosis	Müller et al. (2017)
BLOOD	Human Leukaemia; CEM (acute lymphoblastic), HEL-92 (erythroblastic), HL60 (acute promyelocytic), MOLT-4 (acute lymphoblastic) and PBMCs	Δ^9^-THC	0–100 µM	Cell death via activation of MAPK	Powles et al. (2005)
	Human Leukaemia, Jurkat, MOLT-4 and murine lymphoma, EL-4	CBD, SR141716A, SR144528, CAPZ	0- 10 µM	Significant reduction in cell viability and apoptosis through the CB_2_ receptor	McKallip et al. (2006)
	Human Myeloma, U266, U266-LR7, RPMI, RPMI-LR5, MM1.S, MM1.R	WIN 55,212–2	5–50 µM	Apoptosis	Barbado et al. (2017)
	Human T acute lymphoblastic leukaemia, Jurkat	CBD	0.01–10 µM	Reduction in cell viability and cell cycle changes	Kalenderoglu et al. (2017)
SKIN	Melanoma, A375, MelJuso and murine melanoma, B16	Δ^9^-THC, WIN-55,212–2, SR141716, SR144528	0.5–1 µM	Reduction in cell viability, angiogenesis, and metastasis via CB receptors	Blázquez et al. (2006)
	Human Melanoma, CHL-1, A375, SK-MEL-28BD	Δ^9^-THC, CBD	0–10 µM	Decrease in cell viability	Armstrong et al. (2015)
	Murine squamous, non-melanoma skin cancer; JWF2	AEA, AMG9810, AM251, AM630	2.5- 40 µM	Reduction in cell viability and apoptosis via ER stress	Soliman et al. (2016)
	Human renal carcinoma, 786-O, SMKT-R2, SMKT-R3, Caki-2, RCC-6, 769-P, Caki-1, ACHN	WIN 55,212–2, JWH-133, SR141716A, AM630	0–25 µM	Reduction in cell proliferation and induction of apoptosis	Khan et al. (2018)
	Human ovarian cancer, SKOV-3	CBD	10–50 µM	Inhibition of cell proliferation	Fraguas-Sánchez et al. (2020)
	Rat Adrenal Gland; PC12 cells	DHA-DA, AEA	0–80 µM	NOS activation, increased Ca^2+^ signalling leading to apoptosis via GPR55 activation	Akimov et al. (2021)

MET-AEA (methanandamide, non-hydrolyzable analogue of AEA), AEA (anandamide), DHA-DA (*N*-docosahexaenoyl dopamine), AM251 (CB_1_ antagonist), HU-210 (CB_1_ agonist), JWH-015 (CB_2_ agonist), JWH-133 (CB_2_ agonist), WIN 55,212–2 (CB_1_ agonist), SR141716 (CB_1_ inverse agonist), SR144528 (CB_2_ inverse agonist), N-oleoylethanolamine (NOE) (acidic ceramidase inhibitor), LY294002 (PI3K inhibitor), PD98059 (ERK inhibitor), PBMCs (peripheral blood mononuclear cells), AM630 (CB_2_ antagonist), GW9662 (PPAR-γ antagonist), GSK066 (PPARβ/δ antagonist), GSK501516 (PPARδ antagonist), AMG9810 (TRPV1 antagonist)

**Table 2:** Pre-clinical in vivo studies encompassing various cannabinoids in cancer models

	In vivo model	Cannabinoid (s)	Observed changes	References
PDAC	Immunodeficient nude mice and human PDAC cell lines MIA PaCa-2, PANC-1, Capan-2, BxPC-3	Δ^9^-THC, JWH-133, WIN-55,212–2	Reduction in growth of tumour and induction of apoptosis via activation of the p8-ATF-4TRB3 proapoptotic pathway	Carracedo et al. (2006)
	KPC PDAC mouse model (mutations in KRAS, PanIN, TP53) mice with homozygous deletion of GPR55 created KPCG strain	CBD	KPC mice treated with combination of CBD and GEM survived longer than vehicle or GEM alone	Ferro et al. (2018)
BRAIN	Athymic female CD-1 nude mice and human glioma U87, U373 cell lines	CBD, SR141716, SR144528	CBD significantly inhibited the growth of tumours	Massi et al. (2004)
	Nude mice and human astrocytoma U87MG	Δ^9^-THC	Autophagy and apoptosis	Salazar et al. (2009)
	Female C57BL/6 and murine glioma GL261	CBD, Δ^9^-THC (Pure and BDS)	Triple combination of CBD, Δ^9^-THC and irradiation significantly reduced tumour growth	Scott et al. (2004)
	Female Athymic (nu/nu) mice and human glioblastoma U251 and primary glioma stem cells 3832, 387	CBD	Increase in the survival rate of mice bearing GSC xenografts	Singer et al. (2015)
	Immunodeficient (NOD/SCID) mice and human neuroblastoma SK-N-SH cell lines	Δ^9^-THC, CBD	Reduction in the growth of tumours and increase in activated caspase-3	Fisher et al. (2016)
	Nude mice and human glioblastoma U87MG cell line	Δ^9^-THC, CBD	Reduction in tumour growth	López-Valero et al. (2018)
	Nude mice and U87, T98 cell lines	Δ^9^-THC, CBD	Reduction in tumour growth more significant when combined with temozolomide (TMZ)	Torres et al. (2011)
	Nude mice and U87, T98 cell lines	Δ^9^-THC, SR141716, SR144528	Silencing of Mdk sensitizes cannabinoid resistant tumours to Δ^9^-THC anticancer action, although no effect on tumour growth	Lorente et al. (2011)
BREAST	Female adult BALB/c and SCID-NOD mice and murine mammary carcinoma 4T1	Δ^9^-THC	Increase in tumour growth and metastasis due to inhibition of specific antitumor immune response	McKallip et al. (2005)
	Male athymic mice and human breast adenocarcinoma, MDA-MB-231, MCF-7	Δ^9^-THC, CBD, CBG, CBC, SR141716A, SR144528	CBD inhibited tumour growth and reduced lung metastasis	Ligresti et al. (2006)
	Male C57BL/6 N mice and murine mammary carcinoma, TSAE-1	Met-F-AEA, SR141716A	Reduction of metastatic nodes in mice	Santoro et al. (2009)
	Female adult CD1 nude mice and human breast adenocarcinoma, MDA-MB-231	SR141716	Reduction in tumour volume	Sarnataro et al. (2005)
	Severe combined immunodeficient CB-17 mice and human breast adenocarcinoma, MDA-MB-231/luc/486	WIN 55,212–2, JWH-133	40–50% reduction in tumour burden, 65–80% reduction in lung metastases	Hirao-Suzuki et al. (2020)
	Female BALB/cfC3H mice and murine mammary carcinoma 4T1	CBD	Significant reduction of primary tumour mass and size and lung metastatic foci	Shrivastava et al. (2011)
	Female BALB/c and FVB mice and murine mammary carcinoma 4T1	CBD	Reduction in the growth of tumours and vascularity and inhibition of lung metastasis	Grimaldi et al. (2006)
	Female BALB/cfC3H mice and murine mammary carcinoma 4T1	JWH-015	Significant reduction in primary tumour burden and metastasis	McAllister et al. (2011)
COLORECTAL	Male C57BL/6 N mice, chemically induced colon cancer	SR141716	Inhibition of tumour growth and reduction in ACF induced colon cancer	Santoro et al. (2009)
	Male athymic (nu/nu) mice and human hepatocellular carcinoma, HepG2 and HuH-7	Δ^9^-THC, JWH-015, SR-141716, SR144258	Reduction of tumour growth and ascites	Vara et al. (2011)
	Male ICR mice and human colorectal carcinoma, Caco-2 and HCT116	CBD	Reduction in ACF, polyps and tumour formation in AOM model	Aviello et al. (2012)
	Male ICR and athymic nude female mice and human colon adenocarcinoma, Caco-2 and HCT 116	CBG, AM-251, AM-630	CBG inhibited colon cancer growth	Borelli et al. (2014)
	Male ICR and athymic nude mice and human colorectal carcinoma, DLD-1 and HCT 116	CBD, CBD BS	Reduction of AOM induced preneoplastic lesions and overall tumour growth	Romano et al. (2014)
	Female SCID mice and human colon cancer, HCT116 and SW48	SR141716	Significant reduction in tumour growth Destabilization of the nuclear localization of β-Catenin	Proto et al. (2017)
PROSTATE	Male MR-1 nude mice and prostate carcinoma, LNCaP, 22RV1, DU-145 and PC-3	CBC, CBD, CBG, CBN, CBDA, CBGA, CBDV, CBGV, THC, THCA, THCV, THCVA BDS	Reduction of the LNCaP xenograft growth	De Petrocellis et al. (2013)
	Male athymic nude-FOxn1 (nu/nu) mice and human prostate cancer LNCaP	WIN 55,212–2, SR-141716, SR-144528	Reduction in rate of growth and size of tumours	Morell et al. (2016)
LUNG	Male C57BL/6 (H-2^b^) and BALB/c mice (H-2d) and murine Lewis/alveolar cell lung carcinoma	Δ^9^-THC, SR141716, SR144528	Increase in the growth of the 3LL and L1C2 tumors in vivo	Zhu et al. (2000)
	SCID CB-17 mice and human NSCLC, EGF-induced, A549, SW-1573	Δ^9^-THC, WIN 55,212–2, JWH-133	Inhibition of tumour growth and reduction in lung metastasis	Preet et al. (2008)
	Female NMRI (nu/nu) mice and human Lung adenocarcinoma, A549, H460	CBD, AM-251, AM-630	Reduction in tumour growth	Ramer et al. (2013)
	FVB mice and human Non-small cell lung cancer (NSCLC); A549, CALU1. Murine ED1	JWH-015, SR144528	Reduction in tumour growth and metastatic lesions	Ravi et al. (2016)
BLOOD	Female adult mice C57BL/6	CBD, SR141716A, SR144528	Reduction in tumour growth	McKallip et al. (2006)
SKIN	C57BL/6 nude mice and murine melanoma, B16 cell line	Δ^9^-THC, WIN-55,212–2, SR141716, SR144528	Decrease in tumour growth, proliferation, angiogenesis, and metastasis	Blázquez et al. (2006)
	Male athymic nude (nu/nu) mice and human melanoma, CHL-1, A375, SK-MEL-28BD cell lines	Δ^9^-THC, CBD	Reduction in tumour growth	Ramer et al. (2013)
	NOD/scid/IL-2R gammae null (NSG) mice and human myeloma, U266, U266-LR7, RPMI, RPMI-LR5, MM1.S, MM1.R cells	WIN 55,212–2	Reduction in tumour growth	Barbado et al. (2017)
	Female C57B6 mice and human rhabdomyosarcoma, RD, JR1, RH6, RH2 (ERMS) and RH30, RH4, RH41, RH3, and RH28 (ARMS)	AM251	Abrogates lung metastasis formation	Marshall et al. (2011)

### Pancreatic adenocarcinoma

#### In vitro

A study analysing the in vitro effects of synthetic receptor agonists of CB_1_ and CB_2_, WIN55, 212–2, ACEA and JWH-015 found they each induced a high level of apoptosis of MIA PaCa-2 cells (Console-Bram et al. 2014). The same study showed that a CB_1_ antagonist, *N*-(piperidin-1-1yl)-5-(4-iodophenyl)-1-(2,4-dichlorophenyl)-4-methyl-1H-pyrazole-3-carboxamide (AM251), induced apoptosis and transcriptional changes of the genes involved in the janus kinase/signal transducers, activators of transcription signalling network (JAK/STAT) and MAPK signalling pathways in the MIA PaCa-2 pancreatic cancer cell line through activation independent of the CB_1_ receptor-independent pathways (Fogli et al. 2006). AM251, which expresses molecular similarities with cyclo-oxygenase-2 (COX-2) inhibitor celecoxib, demonstrated a synergistic interaction with 5-fluorouracil (5-FU) increasing their anti-cancer activity when administered in appropriate ratios as demonstrated by a combination index of 0.52 (Fogli et al. 2006).

Dando et al. report arachidonoyl cyclopropylamide (ACPA) and GW, CB_1_ and CB_2_ selective agonists, respectively, inhibited proliferation and invasion of PANC-1 cells (Dando et al. 2013). Activation of the receptors via cannabinoid receptor agonists showed an elevation in 5′ adenosine monophosphate-activated protein kinase (APMK) activation via a ROS-dependent increase of AMP/ATP ratio promoting cell autophagy and subsequent inhibition of cell growth (Dando et al. 2013; Brandi et al. 2013). ∆^9^-THC has been shown to induce a reduction in cell viability via apoptosis in a dose-dependent manner, specifically via the de novo synthesized ceramide up-regulation of the p8 and ATF-4, TRIB3 ER stress genes in MIA PaCa-2 and PANC-1 cells (Carracedo et al. 2006). The p8 protein has been shown to increase with ceramide treatment and potentiates anticancer effects (Javid et al. 2016). In support of this, MIA PaCa-2 cells treated with ∆^9^-THC caused an increase in p8 mRNA levels in vitro. Knockdown of the p8 gene prevented apoptosis by ∆^9^-THC in these cells (Carracedo et al. 2006). In addition to p8 and TRIB3 stress-related genes, further ER stress-inducing genes have been identified and associated with apoptosis, such as CHOP and ATF-4, where mRNA levels were elevated following ∆^9^-THC treatment (Ohoko et al. 2005).

Cannabinoids in combination with chemotherapy agents have shown promising results in pancreatic cancer cell line studies. One study reported the increase in gemcitabine activity by synergism with CB_1_ and CB_2_ receptor ligands by a NF-κß-dependent mechanism (Donadelli et al. 2011). This synergistic inhibition of tumour growth was most marked in gemcitabine-resistant cell lines (Donadelli et al. 2011). Gemcitabine increased cannabinoid-induced autophagy through a ROS-mediated mechanism and cannabinoids enhanced the apoptotic effect of gemcitabine (Donadelli et al. 2011). Ferro and co-workers reported the anticancer effects of blocking the putative GPR55 receptor in pancreatic cancer cells via CBD. A cross between GPR55 homozygous knockout and mice which do not harbour the TP53 mutation did not reveal any statistical difference in survival. Investigators analysed the possible role that p53 may play in regulating GPR55. In pancreatic ductal adenocarcinoma cell lines, they report a negative regulation of GPR55 with TP53 status, where overexpression of wild-type p53 in the AsPC-1 cell line (harbouring a TP53 mutation) caused a reduction in GPR55 expression. Further analysis revealed the negative regulation was through modulation of the micro-RNA miR34b-3p. Pharmacological inhibition of GPR55 via CBD in various pancreatic cell lines, inhibited anchorage-dependent growth. Treatment with CID16020046 (CID), an antagonist of GPR55, revealed similar results in AsPC-1 and HPFA-II and cell cycle arrest at the G_1_–S phase in PANC-1 and HPFA-II in a dose-dependent manner. Cyclin D1, activation of tumour-suppressor protein (RB) was also reduced in CBD treatment and an inhibition of MEK/ERK and ERK-dependent pathways was also observed. The study demonstrates a novel pathway by which gemcitabine may be potentiating anticancer effects through inhibiting GPR55 via CBD antagonization (Ferro et al. 2018).

#### In vivo

Administration of ∆^9^-THC at 15 mg/kg/day into a xenograft model of MIA PaCa-2 pancreatic tumour growth showed a reduction in the tumour burden (Carracedo et al. 2006). A synthetic cannabinoid, WIN55, 212–2 was found to increase the expression of downstream targets of the ER stress-related pathway involved in apoptosis in pancreatic cancer in comparison to healthy controls, demonstrating apoptotic selectivity effect of cannabinoids to cancer cells (Carracedo et al. 2006).

The role of other cannabinoid receptors including GPR55 has been speculated to be involved in regulating many cancer types including pancreatic cancer. A study by Ferro et al. revealed genetic ablation of GPR55 in a KPC mouse model of pancreatic ductal adenocarcinoma (PDAC) significantly prolonged survival and KPC mice treated with CBD and gemcitabine as a combination treatment survived three times longer than control or gemcitabine single treatment (Ferro et al. 2018). Immunohistochemistry analysis of the tumours revealed CBD inhibition of GPR55 affected signalling pathways involved in gemcitabine resistance. CBD was able to counteract the effect of gemcitabine on ERK phosphorylation and downregulated the enzyme’s ribonucleotide reductases 1 and 2 (RRM1/2), a marker for gemcitabine resistance (Ferro et al. 2018). In line with this, gemcitabine-treated tumours from KPC mice expressed high levels of RRM1 and reduced levels were observed in KPCG mice upon treatment with CBD (Ferro et al. 2018). The counteractions of CBD on gemcitabine only occurred when both drugs were administered together, suggesting synergistic effects of CBD on gemcitabine’s mode of action in vivo (Ferro et al. 2018). Donadelli et al. also reported an enhanced effect with combination therapy. CB_1_ antagonist, Rimonabant, combined with gemcitabine reduced tumour growth when compared to single therapy in vivo (Donadelli et al. 2011)*.* An increase in ROS and autophagy pathways were observed which may explain the synergistic effects they observed (Donadelli et al. 2011)*.*

The translation of preclinical data to the clinic remains to be somewhat unclear as many factors in cannabinoids pharmacokinetics, bioactivity and efficacy remain undetermined (Ladin et al. 2016; Millar et al. 2018). In addition, their low aqueous solubility and poor stability (sensitivity to light, temperature and oxidation) make developing effective formulations a problem (Fraguas-Sánchez et al. 2020). The route of cannabinoid administration remains uncertain as the oral bioavailability is very low and is subject to a significant first-pass effect in the body (Millar et al. 2018). Therefore, alternative routes of administration are required, although it has been reported that intratumour (IT) administration of low doses of cannabinoids has improved efficacy of the drug as well as survival (Ngwa et al. 2017, 2018; Yasmin-Karim et al. 2018). Successful administration has been reported when cannabinoids were combined with radiotherapy in treating pancreatic cancer (Yasmin-Karim et al. 2018).

A recent study has reported the use of CBD and ∆^9^-THC inhibited proliferation of pancreatic cancer and stellate cells. PDL-1 (a key target for immune checkpoint blockade) expression was reduced in mice tumours via the PAK-1-dependent pathway (p-21 activated kinase 1) activated by Kirsten rat sarcoma (KRAS). Their findings suggest a novelty for the cannabinoids in which KRAS, an undruggable target expressed in many lethal cancers can be supressed through targeting PAK1 and the suppression of PDL-1 could be enhanced for immune checkpoint blockade therapy in pancreatic cancers (Yang et al. 2020).

### Brain cancer

#### In vitro

Investigation into human glioma cell lines U87 and U373 administered with CBD led to a decrease in mitochondrial oxidative metabolism, cell viability and antiproliferative effects correlated to induction of apoptosis (Massi et al. 2004). Solinas et al. investigated CBD in U87-MG and T98G glioma cell lines and reported inhibition of cell proliferation and invasiveness, a downregulation of ERK and Akt signalling and a decrease in the hypoxia-inducible factor HIF-1α expression (Solinas et al. 2013). In the following neuroblastoma cell lines, SK-N-SH, IMR-32, NUB-6 and LAN-1, CBD and ∆^9^-THC treatment induced antitumorigenic activity by decreasing cell viability and invasiveness, arrest of the cell cycle at the G_1_/G_0_ phase and an increase in activated caspase-3, albeit CBD was more potent in these effects when compared to ∆^9^-THC (Fisher et al. 2016). Salazar et al. investigated ∆^9^-THC in the astrocytoma cell line U87MG and in vivo where they report autophagy induction via the upregulation of p8 leading to apoptosis and inhibition of Akt and mTORC1 (Salazar et al. 2009).

A recent study has reported in the following human glioma cell lines A172, U251, U87 MG, U118 MG and LN18, CBD induced autophagic rather than apoptotic cell death. Specifically, CBD caused mitochondrial dysfunction and lethal mitophagy arrest mechanistically via TRPV4 with an influx of calcium (Huang et al. 2021). Further analysis revealed ER stress and in particular the ATF4-DDIT3-TRIB3-AKT-MTOR axis downstream of TRPV4 was involved in CBD’s mitophagy effect. Combination of CBD and temozolomide (TMZ) in neurosphere cultures and mouse models conveyed synergistic effects in reducing tumour burden and improving survival rates (Torres et al. 2011). Their findings suggest a novel TRPV4-CBD-mitophagy pathway in glioma and combination of CBD and TMZ as a potential to explore in future clinical studies. Additionally, Vrechi and colleagues show CBD stimulates autophagy signal transduction via crosstalk of ERK1/2 and AKT kinases and that CBD-induced autophagy was reduced in presence of CB receptors and TRPV1 receptor antagonists, AM251, AM630 and capsazepine in neuroblastoma and murine astrocyte cell lines (Vrechi et al. 2021).

Kolbe et al. recently investigated the effects of cannabinoids in glioblastoma multiforme (GBM) cells derived from primary human tumour samples and to identify possible receptors involved. Their findings revealed ∆^9^-THC reduced the number of Ki67 immuno-reactive nuclei, through GPR55. Their findings suggest that the sensitivity of cannabinoids and receptor-dependent signalling pathways should be considered to reflect the heterogeneity amongst GBM forms which is critical for when evaluating this translationally to clinic (Kolbe et al. 2021). Mutation-driven cancers are important to take into account when tailoring specific treatments. In a recent paper, Ellert-Miklaszewska et al. investigated the use of synthetic cannabinoids in GBM which have frequent TP53 or PTEN genetic defects rendering it from chemotherapy treatments. Their experimental work showed synthetic cannabinoids not only reduce tumour cells but that p53 could also act as an activator or inhibitor of autophagy and apoptosis and this depends on subcellular localisation and the mutant variant of p53 (Ellert-Miklaszewska et al. 2021).

#### In vivo

In a glioma mouse model treated with CBD daily at 0.5 mg/mouse, Massi and colleagues reported a significant reduction in xenografted human U87 tumour growth in vivo (Massi et al. 2004). A further study investigating CBD’s action in tumours from derived glioma stem cells (GSCs) which known to be resistant to therapies, reported in vivoan increase in the production of ROS leading to the inhibition of cell survival and an increase in the survival rate of mice bearing the GSC xenograft (Singer et al. 2015). They also observed activation of the p-p38 pathway and a downregulation of stem cell regulators including Sox2, Id-1 (a transcription factor involved in cell growth, senescence and differentiation) and p-STAT3 which inhibited the self-renewal of the cells (Singer et al. 2015). Although CBD inhibited glioma progression, a fraction of therapeutic resistance to CBD in a subset of glioma cells was due to the upregulation of antioxidant response genes (Singer et al. 2015). SK-N-SH neuroblastoma cell line induced in nude mice treated with CBD and ∆^9^-THC led to a reduction in tumour burden and an observed increase in activated caspase-3 (Fisher et al. 2016). Various forms of cannabinoids have been trialled and tested to measure the most efficacious form for oncological effects and these include a pure (P) form versus a botanical drug substance (BDS) which is an active form of the drug that has been cultivated usually available as a powder, tablet or elixir. In a study by Scott et al. using P and BDS forms for both CBD and ∆^9^-THC, they report efficacious activity for CBD-P in comparison to CBD-BDS and vice versa for ∆^9^-THC (Scott et al. 2014). As discussed earlier in their in vitrofindings, they report similar outcomes in their orthotopic murine model of glioma and in particular they observed a significant decrease in tumour volumes when both cannabinoids were administered with irradiation, *p* < 0.001 (Scott et al. 2014). These findings support the anticancer effects of cannabinoid treatment in glioma as a single therapy and also as an addition in combination treatment.

Cannabinoids share the common anticancer effect of apoptosis in their mode of action; however, it has also become apparent that autophagy is also involved. The process of apoptosis and autophagy interplay, where the survival function of autophagy negatively regulates apoptosis and inhibition of apoptosis blocks autophagy (Marino et al. 2014). Salazar and co-workers investigated ∆^9^-THC in a murine model of astrocytoma and found that autophagy is upstream of apoptosis in cannabinoid-induced cell death as shown by blocking autophagy, prevented cannabinoid-induced apoptosis (Salazar et al. 2009). ∆^9^-THC induced the effects of stimulation of ceramide synthesis de novo, ER stress, upregulation of p8 and TRIB3, phosphorylation of eIF2α on Ser51 via the activation of the CB_1_ receptor (Salazar et al. 2009). A human glioblastoma-induced murine model investigating GICs (glioma initiating cells; a subpopulation of cells responsible for the aggressiveness of GBM) was treated with ∆^9^-THC, CBD and TMZ in varying combinations. They reported an effective tumour reduction when CBD and ∆^9^-THC with TMZ were co-administered and that treatment with a high ratio of CBD was most efficacious (López-Valero et al. 2018).

### Breast cancer

#### In vitro

McKallip et al. investigated the effects of ∆^9^-THC in human breast cancer cell lines MDA-MB-231, MCF-7 and mouse mammary carcinoma 4T-1. They reported a low expression of cannabinoid receptors; CB_1_ and CB_2_ in these cell lines. ∆^9^-THC did not affect cell viability in MCF-7 and 4T-1 cell lines but increased the size of a 4T1 primary tumour and enhanced metastasis in vivo. ∆^9^-THC exposure caused an increase in IL-4 and IL-10 cytokines and suppression of cell-mediated Th1 response by enhancement of Th2 cytokines due to upregulation in Th2-related genes. These findings suggest exposure to ∆^9^-THC may increase susceptibility to breast cancer which does not express cannabinoid receptors and is resistant to ∆^9^-THC-induced apoptosis (McKallip et al. 2005). In another study by Caffarel and colleagues ∆^9^-THC was investigated in the following human breast cancer cell lines; MCF-7, EVSA-T, MDA-MB-231, MDA-MB468, T-47D and SKBr3. They reported a reduction in human breast cancer cell proliferation by arrest of the cell cycle at the G_2_–M phase via down-regulation of the cyclin-dependent kinase (CDK1 or Cdc2) protein and an induction of apoptosis via the CB_2_ cannabinoid receptor which was highly expressed in the EVSA-T cell line. CB_2_ expression was also found to be correlated with tumours that had a low response to conventional therapies and that were also positive for certain prognostic markers including oestrogen, progesterone receptors and the presence of ERBB2/HER-2 oncogene. The psychotropic effects of cannabinoids are mediated via the CB_1_ rather than CB_2,_ suggesting a cannabinoid therapy that would target the CB_2_ receptor would be beneficial (Caffarel et al. 2006). In a follow-up study investigating the ∆^9^-THC antiproliferative mechanism, exposure to ∆^9^-THC upregulated JunD expression, a proto-oncogene which belongs to the AP-1 transcription factor family, in the tumour cells. In addition, they also identified the involvement of the cyclin-dependent kinase inhibitor p27 and testin (a tumour-suppressor gene) as candidate targets of JunD. Stress protein p8, however was involved in ∆^9^-THC antiproliferative action in a JunD-independent manner, suggesting a multimodal mechanism of action (Caffarel et al. 2008).

In an interesting report by Blasco-Benito et al., they found ∆^9^-THC was able to disrupt the HER2–CB_2_R complex by selective binding to CB_2_R. Additionally, they concluded the antitumour efficacy of a botanical drug preparation to be more potent than pure ∆^9^-THC for both cell lines and animal studies (Blasco-Benito et al. 2019). Ligresti et al. investigated the anticancer properties of plant-based cannabinoids including CBD, CBG, CBC, CBDA and ∆^9^-THCA in addition to assessing the use of enriched CBD or ∆^9^-THC cannabis extracts over pure cannabinoids (Ligresti et al. 2006). Within the breast cancer cell lines, MDA-MB-231 and MCF-7, treated with the above cannabinoids, CBD was the most potent in its antiproliferative activity (Ligresti et al. 2006). They also report CBD mediated its apoptotic effects via the following routes: the direct or indirect activation of the receptors CB_2_ and TRPV1, receptor-independent elevation of intracellular Ca^2+^ and ROS generation (Ligresti et al. 2006).

Synthetic agonists or antagonists of cannabinoid receptors have been used to study the role of the ECS in cancer signalling and growth. Sarnataro and co-workers investigated the effects of Rimonabant, a CB_1_ antagonist, in the invasive human breast cancer line MDA-MB-231 and in the less-invasive lines, T47D and MCF-7 (Sarnataro et al. 2006). Treatment with Rimonabant caused antiproliferative effects characteristic of G_1_–S-phase cell cycle arrest accompanied by a downregulation in cyclins D and E with associated upregulation of cyclin-dependent kinase inhibitor p27^KIP1^. No observed apoptosis or necrosis occurred in vitro (Sarnataro et al. 2006). Additionally, within the invasive cells, these effects were found to be associated with lipid raft/caveolae as previously shown by the group (Sarnataro et al. 2005). Rimonabant caused complete displacement of the CB_1_ receptor from lipid rafts and the depletion of cholesterol by methyl-β-cyclodextrin (MCD) prevented these effects (Sarnataro et al. 2006). In cells overexpressing the CB_1_ receptor, Rimonabant inhibited MAPK signalling and decreased ERK1/2 activity (Sarnataro et al. 2006). Pre-treatment with MCD before Rimonabant administration caused a depletion in cholesterol and this reverted the inhibitory effects on ERK1/2 via Rimonabant, suggesting an interplay between the CB_1_ receptor and lipid raft motility in breast tumour growth (Sarnataro et al. 2006). JWH-015, an agonist of the CB_2_ receptor, in human MCF-7 mammary carcinoma cells reduced viability by inducing apoptosis independent of G_αi_ signalling or by pharmacological blockade of CB_1_, GPR55, TRPV1 or TRPA1 receptors and instead these effects were calcium-dependent and caused changes in MAPK/ERK signalling (Hanlon et al. 2016).

CBD has also been shown to downregulate Id-1 in the aggressive human breast cancer line MDA-MB-231 through modulation of ERK and ROS pathways leading to a decrease in Id-1 expression and also upregulated Id-2 (a transcriptional regulator) (McAllister et al. 2011). Shrivastava et al. observed a complex interplay between apoptosis and autophagy in CBD-treated invasive breast cancer cells, MDA-MB-231 (Shrivastava et al. 2011). In particular, CBD induced ER stress which led to the inhibition of AKT and mTOR signalling in vitroindicated by low levels of phosphorylated cyclin D1, mTOR and 4EBP1 (Shrivastava et al. 2011). Further analysis revealed CBD inhibited the association between beclin1 (central role in autophagy) and BCL-2 known to inhibit autophagy through cleavage of Beclin-1 and enhanced the interaction between Beclin-1 and Vps34 favouring autophagy (Shrivastava et al. 2011). Electron microscopy revealed morphological changes to MDA-MB-231 CBD-treated cells which included nuclear condensation, margination, increased vacuolization, decrease in intracellular organelles and enlarged mitochondria evident of apoptotic activity (Shrivastava et al. 2011). They hypothesized that the event changes in inducing autophagy may also cause apoptosis as the cleavage product from Beclin-1 translocates to the mitochondria and induces cytochrome C (Shrivastava et al. 2011). These observations and hypothesis suggest CBD may be able to control the complex interplay between autophagy and apoptosis in these breast cancer cells (Shrivastava et al. 2011). CBD also increased ROS levels and blockage of ROS inhibited apoptotic and autophagy pathways (Shrivastava et al. 2011). These effects were independent of cannabinoid and vanilloid receptor activation (Shrivastava et al. 2011).

Many drugs have failed in clinics for many of the aggressive cancers due to the recalcitrant TME. The TME plays a major role in contributing to the growth and invasion of cancer and in particular tumour-associated macrophages (TAMs) which are a class of immune cells contributing to the immunosuppressive TME through interchange of its two forms: M1 (anti-tumorigenic) and M2 (pro-tumorigenic) (Lin et al. 2019). Elbaz and colleagues investigated CBD in triple-negative breast cancer (TNBC) cell lines SUM159, MDA-MB-231-SCP2, MVT-1, 4T1.2 and in murine leukaemia RAW264.7. They observed CBD inhibited EGF-induced proliferation and chemotaxis in the cell lines, activated EGFR, ERK, Akt, and NF-κß pathways in addition to inhibition of matrix metallopeptidase 2 and 9 (MMP2 and MMP9) secretion (Elbaz et al. 2015). A cancer education experiment (conditioned media from CBD-treated cancer cells) showed a significant reduction in the number of migrated RAW 264.7 cells towards this medium which also contained lower levels of granulocyte–macrophage colony-stimulating factor (GM-CSF) and chemokine ligand 3 (CCL3) cytokines, crucial for macrophage recruitment and activation (Elbaz et al. 2015). They observed a reduction in tumour growth and metastasis and inhibition of the recruitment of total and M2 macrophages to the stroma of the primary tumour and secondary lung metastasis (Elbaz et al. 2015).

Cannabinoid’s effect on ER has been evident in many cancer studies, however, the exact mechanism by which this occurs remain elusive. In a recent study by de la Harpe et al., they found CBD selectively targeted MCF7 cells via oxidative stress-induced ER stress and UPR (unfolded protein response) activation, and these effects were caused by calcium influx via the TRPV1 receptor as opposed to MDA-MB-231 cells. This suggests the difference in CBD treatment was dependent on localization of TRPV1 (de la Harpe et al., 2021).

#### In vivo

One of the factors to consider in cannabinoid treatment is the abundance of cannabinoid receptors in the tissue of interest. In a study investigating the effects of ∆^9^-THC in a murine model of mammary carcinoma, it was found that the murine mammary carcinoma cell line 4T1 first did not express detectable levels of the cannabinoid receptors CB_1_ and CB_2_ and second, these cells were resistant to the cytotoxicity of ∆^9^-THC. They also show treatment with ∆^9^-THC led to an increase in tumour growth and metastasis due to an increase in production of IL-4 and IL-10 which suppressed the cell-mediated Th1 response by enhancing Th2-associated cytokines (McKallip et al. 2005). This finding was supported by the injection of anti-IL-4 and anti-IL-10 monoclonal antibodies which partially reversed the immune suppression of ∆^9^-THC in 4T1 cells (McKallip et al. 2005).

A study investigating the effects of the endogenous cannabinoid, Met-F-AEA (a metabolically stable anandamide analogue) in a highly invasive murine breast cancer model reported a significantly reduced amount and size of metastatic nodes and this effect was antagonized by the selective CB_1_ antagonist Rimonabant (Grimaldi et al. 2006). Molecular interrogation in treated cells with the endogenous cannabinoid caused a decrease in tyrosine phosphorylation of focal adhesion kinase (FAK) and steroid receptor coactivator (Src) and these effects were mitigated by Rimonabant (Grimaldi et al. 2006). They concluded CB_1_ receptor agonists by modulating FAK phosphorylation inhibited tumour cell invasion and metastasis and therefore CB_1_ receptor activation may represent a novel therapeutic target for the treatment of breast carcinoma and metastasis (Grimaldi et al. 2006). Rimonabant has also been reported to significantly reduce tumour volume in vivo in the invasive human MDA-MD-231 murine model and this effect occurred via the CB_1_R lipid raft/caveolae-mediated mechanism (Sarnataro et al. 2006).

In a human MDA-MB-231 breast carcinoma xenografted tumour model, both CBD and CBD enriched extract treatment induced apoptosis, inhibited the growth of tumours and metastasis in vivo (Ligresti et al. 2006). CBD has also been shown to modulate transcriptional activity downstream in breast cancer. A study by McAllister and colleagues investigated CBD treatment of a murine model of metastatic breast cancer and found CBD inhibited Id-1 gene expression in the primary tumour and lung metastasis in vivo through modulation of the ERK and ROS pathways (McAllister et al. 2011). Caffarel et al. have shown using a genetically engineered animal model of ErbB2-driven metastatic breast cancer (MMTV-neu mice), ∆^9^-THC and JWH-133 (selective CB_2_ agonist) reduce metastatic progression via AKT pathway inhibition (Caffarel et al. 2010).

Cannabinoids mechanistic actions have been reported to be CB-independent with studies reporting other channels through which they may activate their oncological effects, such as GPR55 or vanilloid channels. Hanlon and co-workers report using JWH-015, a CB_2_ agonist, significantly reduced tumour burden and metastasis of murine mammary carcinoma 4T1 cells in immunocompetent mice and these effects were dependent on calcium and induced changes to MAPK/ERK signalling which were independent of G-protein-coupled signalling, CB or vanilloid receptors (McAllister et al. 2011).

### Other gastrointestinal (GI) cancers

#### In vitro

In a study investigating human colorectal cancer cells using the lines DLD-1, CaCo-2 and SW620, treatment with Rimonabant significantly reduced cell proliferation and induced death. In DLD-1 cells, treatment resulted in G_2_–M-phase cell cycle arrest without inducing apoptosis or necrosis (Aviello et al. 2012). Further investigation revealed an increase in mitotic catastrophe characterized by changes in the following, cyclin B1, PARP-1 (involved in DNA repair) Aurora B (involved in the attachment of the mitotic spindle in prophase), phosphorylated p38/MAPK and Chk1 (checkpoint kinase 1) in a time-dependent manner (Aviello et al. 2012). Rimonabant, can therefore mediate cancer tumour growth via mitotic catastrophe inducing cell-cycle arrest during spindle assembly and DNA-damage checkpoints (Aviello et al. 2012).

In hepatocellular carcinoma cell lines, HepG2 and Huh-7, treatment with ∆^9^-THC and JWH-015 (synthetic CB_2_ receptor agonist) reduced cell viability through activation of the CB_2_ receptor. Autophagy was subsequently induced by the upregulation of TRIB3, stimulation of adenosine monophosphate-activated kinase (AMPK) and Akt/mTORC1 inhibition (Vara et al. 2011).

In human colorectal cell lines, Caco-2 and HCT116, CBD treatment protected DNA from oxidative damage, reduced cell proliferation and increased endocannabinoid levels via CB_1_, TRPV1 and PPARγ (Romano et al. 2014). In addition, CBD treatment of colorectal carcinoma cell line DLD-1, reduced cell proliferation (Macpherson et al. 2014). An interesting study investigated the antiproliferative effects of CBD in Caco-2 cell line in various oxygen environments and found the antitumour effects of CBD to be greater in PhysO_2_ than AtmosO_2_. They conclude that CBD induced a mitochondrial production of ROS in PhysO_2_ cells, suggesting that the cellular redox environment can influence how CBD induced antiproliferative effects in PhysO_2_ to AtmosO_2_ cells (Nallathambi et al. 2018). This study demonstrates the important role microenvironments play in cell cultures when studying the pharmacokinetics and mechanism of drugs. Macpherson and colleagues report the increase in sensitivity to CBD-induced antiproliferative effects through changes to cell energetics, from a drop in oxygen and a loss in mitochondrial membrane integrity in cells under the atmospheric condition to the increase in ROS in mitochondria under low oxygen conditions (Nallathambi et al. 2018).

Purified cannabinoids have been mainly reported in inducing apoptosis, inhibiting proliferation and metastasis in many cancer types, however, other forms such as unheated extracts of the plants have been less studied. Nallathambi and colleagues identified unheated extract fractions (F7: THCA, F3: CBGA) from *C. sativa* which displayed cytotoxic effects in colorectal cancer cell lines, HCT116 and CCD-18Co and adenomatous polyps but reduced activity on normal colon cell lines (Nallathambi et al. 2018). Combination treatment analysed by the Bliss independence model, exhibited more potent cytotoxic effects which included cell cycle arrest, cell death and a reduction in genes involved in the Wnt signalling pathway (Proto et al. 2017).

#### In vivo

Rimonabant treatment in a mouse model of azoxymethane-induced colon carcinogenesis caused a significant reduction in aberrant crypt foci formation, which is a neoplastic precursor to colorectal cancer and additionally observed inhibitory effects with changes to mitotic and DNA-damage checkpoints in their cell lines as mentioned previously (Aviello et al. 2012). Another study investigated the synthetic cannabinoids effects on the Wnt/β-catenin pathway, a signalling pathway involved in the formation of colorectal cancer (Borelli et al. 2014). The administration of rimonabant in HCT116 xenografts caused a significant reduction in tumour growth and destabilized the nuclear localization of β-catenin in vivo by inhibiting the canonical Wnt pathway (Borelli et al. 2014). This study suggests a novel use for cannabinoids in treating colorectal cancer harbouring mutations in β-catenin.

In a murine model of hepatocellular carcinoma, treatment with JWH-015 and ∆^9^-THC, both cannabinoids reduced subcutaneous xenograft growth; however, this effect was not observed when autophagy was pharmacologically inhibited (Vara et al. 2011) indicating the importance of cell death in both cannabinoids reducing tumour burden in vivo. Furthermore, administration of the cannabinoids also led to a reduction in ascites (abnormal build-up of fluid in the abdomen) formation (Vara et al. 2011). In support of the mechanisms observed in the HCC cell lines, Salazar et al. investigated ∆^9^-THC in the astrocytoma cell line U87MG and in vivo where they report autophagy induction via the upregulation of p8 leading to apoptosis and inhibition of Akt and mTORC1 (Salazar et al. 2009).

The effect of CBD in gastrointestinal cancers has also been studied. In a study by Aviello et al., CBD treatment in an azoxymethane (AOM)-induced murine model of colon cancer, reduced aberrant crypt foci, polyps, tumour growth and led to a decrease in expression of inducible nitric oxide synthase (iNOS) and phosphorylated Akt with an upregulation in caspase-3 (Aviello et al. 2012). CBG’s anticancer effect has been observed in colon cancer models. Borelli et al. evaluated the antineoplastic effects in xenograft models of colon cancer and observed a reduction tumour growth, however due to the limitation in the model, they further tested CBG in an AOM colon murine model which mimics the tumour in situ and found CBG completely abolished the formation of aberrant crypt foci and reduced the number of tumours (Borelli et al. 2014). In addition, Romano et al. tested the effects of the BDS form of CBD, which contains a high content of CBD on colorectal cancer growth in both xenograft and AOM models. They also observed a reduction in tumour growth, preneoplastic lesions and polyps (Macpherson et al. 2014).

### Prostate cancer

#### In vitro

∆^9^-THC induced apoptosis in a PC-3 prostate cancer cell line in a dose-dependent manner (Sreevalsan et al. 2011). CBD’s pro-apoptotic nature has been shown to be phosphate-dependent in prostate and colon cancer cells (De Petrocellis et al. 2013). In LNCaP (prostate) and SW480 (colon) cancer cell lines, the growth and mRNA expression of several phosphatases inhibited cannabinoid-induced PARP cleavage (De Petrocellis et al. 2013). De Petrocellis et al. investigated CBD’s effect in prostate carcinoma cell lines; LNCaP, 22RV1 (positive for androgen receptor), DU-145 and PC-3 (negative for androgen receptor). CBD treatment significantly decreased cell viability and potentiated the effects of bicalutamide and docetaxel (standard drugs for treatment of prostate carcinoma) against LNCaP and DU-145 xenograft tumours and when given alone reduced LNCaP xenograft size. CBD administered between 1 and 10 µM induced apoptosis and markers of intrinsic apoptotic pathways (PUMA, CHOP expression and intracellular Ca^2+^). In LNCaP cells, the pro-apoptotic effect of CBD was only partly due to TRPM8 antagonism and was accompanied by down-regulation of AR, p53 activation and elevation of ROS. LNCaP cells differentiated to androgen-insensitive neuroendocrine-like cells were more sensitive to CBD-induced apoptosis (De Petrocellis et al. 2013).

### Gynaecological cancers

#### In vitro

The effects of ∆^9^-THC were also investigated in aggressive endometrial cancer. Zhang et al. report in HEC-1B and An3ca aggressive endometrial cancer cell lines a high level of cannabinoid receptor expression and treatment with ∆^9^-THC inhibited cell viability and motility by inhibiting epithelial-mesenchymal transition (EMT) in addition to down-regulation of the MMP9 gene in inhibiting metastasis. These findings suggest regulation and targeting of the MMP9-related pathways via ∆^9^-THC treatment may inhibit metastasis in this aggressive cancer type (Zhang et al. 2018). A recent study investigated the oncological effects of CBD as a monotherapy and in combination with chemotherapy drugs in ovarian cancer, administered as Poly lactic-*co*-glycolytic acid (PGLA)-microparticles (Fraguas-Sánchez et al. 2020). Their results show the combination of paclitaxel (PTX) with CBD to be effective in vitro and in ovo (Fraguas-Sánchez et al. 2020). CBD administered as microparticles was more efficacious than in single solution and in ovo, PTX resulted in a 1.5-fold tumour growth inhibition whereas in combination with CBD this increased to a twofold decrease, suggesting a promising therapy to explore in treating ovarian cancer as it provides the advantageous effect of using a lower dose of the antineoplastic drug whilst maintaining the same efficacy (Fraguas-Sánchez et al. 2020).

### Clinical studies

The anticancer effects of cannabinoids have so far been limited to preclinical studies and translation to the clinic has remained stagnant. The first report of the use of cannabinoids on cancer patients was a pilot study that investigated ∆^9^-THC on nine terminal patients with recurrent glioblastoma where standard therapy remained unhopeful as a curative (Guzmán et al. 2006). These patients underwent intracranial administration of ∆^9^-THC, as this route was deemed the safest and patients did not exhibit any of the associated psychoactive effects (Guzmán et al. 2006). In-depth analysis of two patients’ tumours revealed molecular effects associated with cannabinoids antitumour action, which included decreased cell proliferation, stimulation of apoptosis and autophagy (Guzmán et al. 2006). Although positive effects were observed, the small case number hinders any statistically significant conclusions to be drawn from this study.

A recently published completed clinical study investigated the safety and preliminary efficacy of nabiximols oromucosal cannabinoid spray and dose intense (DIT) TMZ in patients with first recurrence glioblastoma (Twelves et al. 2021). The study included an open label arm where patients received nabiximols (*n* = 6) and a randomised, double-blind, and placebo-controlled arm (*n* = 12 and *n* = 9). Up to 12 sprays/days with DIT for 12 months were administered and the safety, efficacy and pharmacokinetics of TMZ were observed. Study reports a 33% of nabiximols and placebo-treated patients were progression free for 6 months and survival at 1 year for nabiximols was 83% and 44% for placebo patients and no effects of nabiximols on TMZ were reported. Although nabiximols spray was tolerable in GBM patients, a major limitation to the study was the small size of enrolled patients, specifically 21 across 9 sites and there was no predetermined power calculation to the study to define the minimum number of patients for statistical power (Twelves et al. 2021). Nevertheless, the observations warrant the need for further clinical trials to help establish safe and efficacious routes of administration, patient sub-stratification and to explore its possible synergistic effects with other antitumour agents as shown in pre-clinical data. Table 3 summarises clinical trials investigating cannabinoids including synthetic versions, CBD and ∆^9^-THC in cancer treatment.

**Table 3:** Overview of clinical trials for investigation of cannabinoids in cancer. Key search terms included: “Cancer and Cannabinoids, Cannabis, Cannabidiol, Tetrahydrocannabinol” (www.clinicaltrials.gov)

Trial name	Conditions	Phase	*n*	Study type	Drug	Location	Status	NCT no.
A Pilot Study of Dronabinol for Adult Patients With Primary Gliomas	Brain Neoplasms|Nausea|Vomiting	I	33	Interventional	Dronabinol	North Carlina, USA	Completed	NCT00314808
A Phase 1 Study of Dexanabinol in Patients With Advanced Solid Tumours	Solid Tumour	I	40	Interventional	Dexanabinol|Other: Cremophor	Leeds/Newcastle/Glasgow	Completed	NCT01489826
A Safety Study of Sativex in Combination With Dose-intense Temozolomide in Patients With Recurrent Glioblastoma	Cancer	I/II	6	Interventional	Sativex	Leeds/Bristol/London	Completed	NCT01812603
A Safety Study of Sativex Compared With Placebo (Both With Dose-intense Temozolomide) in Recurrent Glioblastoma Patients	Cancer	I/II	21	Interventional	Sativex|Placebo	Germany	Completed	NCT01812616
A Pharmacokinetic Study of Single Doses of Sativex in Treatment-induced Mucositis	Head and Neck Squamous Cell Carcinoma	I	10	Interventional	Sativex	London	Terminated	NCT01975688
Assessment of Single Doses of Oral Dexanabinol in Healthy Subjects	Safety|Tolerability|Pharmacokinetics|Cancer	I	40	Interventional	Dexanabinol, Placebo	Nottingham	Completed	NCT02054754
A Study: Pure CBD as Single-agent for Solid Tumor	Solid Tumor	II	60	Interventional	Cannabidiol	Israel	Unknown status	NCT02255292
A Study of Dexanabinol in Combination With Chemotherapy in Patients With Advanced Tumours	Hepatocellular Carcinoma|Pancreatic Cancer	I	112	Interventional	Dexanabinol| Sorafenib| Nab-paclitaxel| Gemcitabine	Switzerland/Germany/Spain	Unknown status	NCT02423239
A Study to Assess the Pharmacokinetic (PK) Properties of SativexÂ® in Patients With Advanced Cancer	Advanced Cancer	I	0	Interventional	Sativex	United Kingdom	Withdrawn	NCT02432612
Study on Cannabinoid Receptor Expression in Gastrointestinal Diseases	Ulcerative Colitis|Crohn's Disease|Colon Cancer	–	31	Observational	N/A	Austria	Completed	NCT02735941
The Effect of Cannabis in Pancreatic Cancer	Neoplasms Pancreatic|Cachexia; Cancer|Cannabis|Appetite Loss|Palliative Medicine|Morbidity|Mortality	II	32	Interventional	THC and CBD Mixture	Denmark	Unknown status	NCT03245658
Tolerability of Cannabis in Patients Receiving Concurrent Chemoradiation for Glioblastoma	Glioblastoma	I	1	Interventional	Cannabis| Temozolomide| Radiation Therapy	New York, USA	Terminated	NCT03246113
Medical Cannabis During Chemoradiation for Head and Neck Cancer	Head and Neck Cancer	–	30	Observational	Cannabis	New York, USA	Recruiting	NCT03431363
TN-TC11G (THC + CBD) Combination With Temozolomide and Radiotherapy in Patients With Newly-diagnosed Glioblastoma	Glioblastoma	I/II	30	Interventional	TN-TC11G| Temozolomide Oral Product| Radiotherapy	Spain	Not yet recruiting	NCT03529448
A Study of the Efficacy of Cannabidiol in Patients With Multiple Myeloma, Glioblastoma Multiforme, and GI Malignancies	Cancer of Pancreas|Cancer of Liver|Cancer of Rectum|Cancer of Colon|Cancer, Gall Bladder|Myeloma Multiple|Glioblastoma Multiforme	I/ II	160	Interventional	Cannabidiol| Bortezomib| Leucovorin| 5-FU| Oxaliplatin| Bevacizumab| Irinotecan| Gemcitabine| Temozolomide	Orlando/Florida, USA	Not yet recruiting	NCT03607643
Cannabis Use in Cancer Patients	Solid Tumor, Adult	–	30	Observational	Cannabis	Colorado, USA	Recruiting	NCT03617692
Pilot, Syndros, Decreasing Use of Opioids in Breast Cancer Subjects With Bone Mets	Bone Metastases|Breast Cancer|Pain	Early I	20	Interventional	Syndros	Arizona, USA	Recruiting	NCT03661892
Pharmacokinetic (PK) and Pharmacodynamics (PD) Study of Ilera Specific Products	Cancer and other ailments	–	10	Observational	Registry|Other: PK microsampling of blood	Philadelphia/ Pennsylvania, USA	Terminated	NCT03886753
Efficacy and Safety of Dronabinol in the Improvement of Chemotherapy-induced and Tumor-related Symptoms in Advanced Pancreatic Cancer	Pancreatic Cancer Non-resectable|Chemotherapy-induced Nausea and Vomiting|Pancreatic Cancer Metastatic	III	140	Interventional	Dronabinol in Oral Dosage Form| Placebo in Oral Dosage Form	Austria	Recruiting	NCT03984214
Dibenzyl Trisulphide (GUINEAHEN WEED) for Stage IV Cancer	Stage IV Prostate Cancer|Stage IV Colon Cancer|Stage IV Breast Cancer|Stage IV Cancer of the Cervix	Early I	104	Interventional	Dibenzyl trisulphide capsules| Placebo	Jamaica	Unknown status	NCT04113096
Effect of Hemp-CBD on Patients With CIPN	Chemotherapy-induced Peripheral Neuropathy|Colorectal Cancer Stage II|Colorectal Cancer Stage III|Breast Cancer|Ovarian Cancer|Uterine Cancer	II	100	Interventional	Hemp-based CBD| Placebo oral tablet	Pennsylvania, USA	Recruiting	NCT04398446
Epidiolex (CBD) in Patients With Biochemically Recurrent Prostate Cancer	Prostate Cancer Recurrent|Prostate Cancer|Prostate Adenocarcinoma	I	18	Interventional	Epidiolex Oral Liquid	Kentucky, USA	Recruiting	NCT04428203

## Conclusion

Plant-based, endogenous and synthetic cannabinoid compounds have shown merits in not only alleviating the unwanted side effects of antineoplastic drug regiments, but have also shown promising evidence in decreasing tumour burden, and one in vivo study so far concludes increasing survival rates in mice. The antitumour effects of cannabinoids trend in modulating processes which include apoptosis and autophagy through first stimulating de novo synthesis of ceramide which induces activation of ER stress-related signalling proteins further leading to the inhibition of the AKT/mTORC1 axis promoting cell cycle arrest and additional mechanisms, such as cell death and aging. Other pathways involved mechanistically are activation of MAPK/ERK signalling through calcium induction. Strategies that would optimize the anticancer effects of cannabinoids through interference of these signalling cross-talks may prove useful for therapeutic intervention. Nevertheless, we found that these effects were reached differently downstream depending on the type of cancer, the dosage of the compound and which receptor/ligands were activated. We also found the co-administration of cannabinoids with chemotherapy drugs enhanced the potency of these effects. These synergistic effects should be targeted for translation to clinical application, especially in cancers which are refractory to chemotherapy. Various extracted forms of cannabinoids from C. *sativa* have shown varying cytotoxic effects which should be explored in more detail in future studies as majority of the evidence originates from studies investigating mainly ∆^9^-THC and CBD’s actions. Whilst the emerging evidence of phytocannabinoid anticancer effects are promising, there remains a paucity of clinical evaluation which must be overcome.
